# Short ORF-Dependent Ribosome Shunting Operates in an RNA Picorna-Like Virus and a DNA Pararetrovirus that Cause Rice Tungro Disease

**DOI:** 10.1371/journal.ppat.1002568

**Published:** 2012-03-01

**Authors:** Mikhail M. Pooggin, Rajendran Rajeswaran, Mikhail V. Schepetilnikov, Lyubov A. Ryabova

**Affiliations:** 1 Institute of Botany, University of Basel, Basel, Switzerland; 2 Institut de Biologie Moléculaire des Plantes du CNRS, Université de Strasbourg, Strasbourg, France; Iowa State University, United States of America

## Abstract

Rice tungro disease is caused by synergistic interaction of an RNA picorna-like virus *Rice tungro spherical virus* (RTSV) and a DNA pararetrovirus *Rice tungro bacilliform virus* (RTBV). It is spread by insects owing to an RTSV-encoded transmission factor. RTBV has evolved a ribosome shunt mechanism to initiate translation of its pregenomic RNA having a long and highly structured leader. We found that a long leader of RTSV genomic RNA remarkably resembles the RTBV leader: both contain several short ORFs (sORFs) and potentially fold into a large stem-loop structure with the first sORF terminating in front of the stem basal helix. Using translation assays in rice protoplasts and wheat germ extracts, we show that, like in RTBV, both initiation and proper termination of the first sORF translation in front of the stem are required for shunt-mediated translation of a reporter ORF placed downstream of the RTSV leader. The base pairing that forms the basal helix is required for shunting, but its sequence can be varied. Shunt efficiency in RTSV is lower than in RTBV. But in addition to shunting the RTSV leader sequence allows relatively efficient linear ribosome migration, which also contributes to translation initiation downstream of the leader. We conclude that RTSV and RTBV have developed a similar, sORF-dependent shunt mechanism possibly to adapt to the host translation system and/or coordinate their life cycles. Given that sORF-dependent shunting also operates in a pararetrovirus *Cauliflower mosaic virus* and likely in other pararetroviruses that possess a conserved shunt configuration in their leaders it is tempting to propose that RTSV may have acquired shunt *cis*-elements from RTBV during their co-existence.

## Author Summary

Ribosome shunting, first discovered in plant pararetroviruses, is a translation initiation mechanism that combines 5′ end-dependent scanning and internal initiation and allows a bypass of highly-structured leaders of certain viral and cellular mRNAs. Here we demonstrate that a similar shunt mechanism has been developed by the RNA picorna-like virus RTSV and the DNA pararetrovirus RTBV that form a disease complex in rice. Leader sequences of the RTSV genomic RNA and the RTBV pregenomic RNA possess a conserved shunt configuration with a 5′-proximal short ORF (sORF1) terminating in front of a large stem-loop structure. Like in RTBV and a related pararetrovirus *Cauliflower mosaic virus*, shunt-mediated translation downstream of the RTSV leader depends on initiation and proper termination of sORF1 translation and on formation of the basal helix of the downstream secondary structure. Given that RTBV-like shunt elements with identical sequence motifs are present in all RTSV isolates but absent in related picorna-like viruses, it is likely that RTSV could have acquired these elements after its encounter with RTBV. Alternatively, the RTSV shunt elements could have evolved independently to adapt to the rice translation machinery. Our study highlights on-going genetic exchange and co-adaptation to the host in emerging viral disease complexes.

## Introduction

Rice tungro disease is a significant constraint for rice cultivation in South and Southeast Asia. It is caused by a synergistic interaction of two viruses, *Rice tungro bacilliform virus* (RTBV) and *Rice tungro spherical virus* (RTSV). Individually these viruses exhibit rather mild symptoms: RTSV causes mild or indistinct symptoms, whereas RTBV infection causes yellowing and reddening of the leaves and results in stunted growth. The RTBV symptoms are accentuated in plants co-infected with RTBV and RTSV. Moreover, RTBV on its own cannot be transmitted from plant to plant, but it can do so with the help of RTSV that encodes an insect transmission factor [1]. This suggests that the two viruses have co-evolved into a unique disease complex, in which partners may have developed not only specialized but also shared mechanisms enabling the complex to establish systemic infection and to accumulate in the same plant tissues in order to be co-transmitted. Indeed, both RTBV and RTSV are phloem-restricted. It can be further suggested that during converging evolution the two viruses may have exchanged or independently developed certain *cis*-acting elements and sequence motifs to adapt to the host cell machinery and to synchronize their life cycles. Our study provides initial evidence for this hypothesis.

RTSV belongs to genus *Waikavirus* in the family *Secoviridae* of picorna-like viruses [2]. Its single-stranded, polyadenylated genomic RNA of 12.4 kb contains one large ORF encoding a viral polyprotein [3]. The polyprotein ORF is preceded with an unusually long leader sequence (514-nt in the type species NC_001632) which has several short ORFs (sORFs) and a high propensity to form stable secondary structure (see below): both features are known to inhibit 5′ end-dependent, scanning-mediated translation initiation on eukaryotic ribosomes [4]. Thus, translation of RTSV genomic RNA may involve either internal ribosome entry or 5′ end-dependent ribosome shunting. An internal initiation mechanism operates in animal picornaviruses that possess long and highly-structured leaders [5] and it is therefore an attractive possibility that plant picorna-like viruses have also evolved an internal ribosome entry site (IRES) to initiate translation. However, so far there is little evidence that viruses of the family *Secoviridae* use internal initiation of translation and the IRES elements identified in short leaders of two distinct viruses from the family *Potyviridae* do not resemble each other and those of animal picornaviruses [6]. Instead, compelling evidence indicates that plant pararetroviruses have evolved a ribosome shunt mechanism, which combines features of 5′ end-dependent scanning and internal initiation, to translate their pregenomic RNAs that all possess long and highly structured leaders [7]–[10].

RTBV is the only member of genus *Tungrovirus* in the family *Caulimoviridae* of pararetroviruses [11]. Its circular double-stranded DNA genome of 8 kbp is transcribed by Pol II into a pregenomic RNA (pgRNA) of more-than-genome length as a poly(A) signal located 195 bp downstream of the transcription start site is recognized efficiently only at its second encounter. The pgRNA is a polycistronic mRNA for three consecutive overlapping ORFs (I, II and III) that are translated by a leaky scanning mechanism [12]. This mechanism operates efficiently owing to the lack of additional AUGs within about 1 kb region between the start codons of ORFs I and III, the feature also conserved in a closely-related badnaviruses (genus *Badnavirus* of the *Caulimoviridae*) which have similar organization of ORFs I–III [13]. Unlike badnaviruses, RTBV has an additional ORF, ORF IV, located downstream of ORF III. This ORF is translated from a spliced version of pgRNA, in which the first sORF of the pgRNA leader is fused to ORF IV [14].

**Figure 1:**
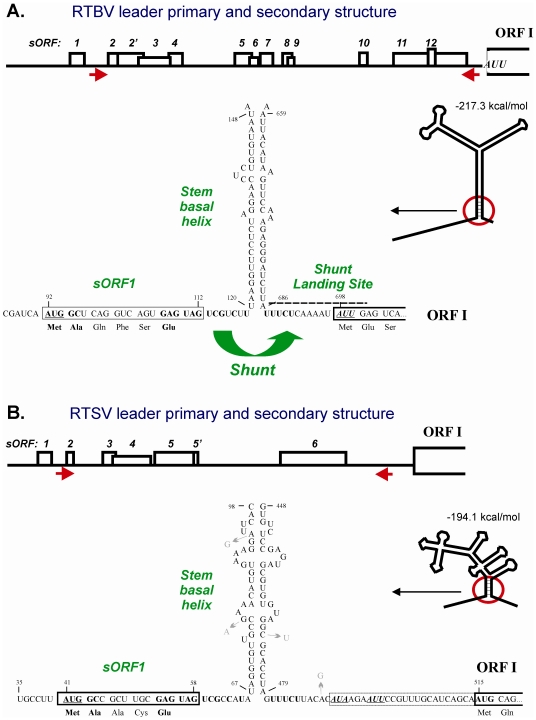
Conserved shunt configurations in the RTBV and RTSV leader sequences. The primary and secondary structures of RTBV (**A**) and RTSV (**B**) leaders preceding the first large viral ORF (ORF I) are shown schematically. Short ORFs within the leaders are indicated by boxes, with internal AUGs indicated by vertical lines. Arrows under the leader line define the ascending and descending arms that form the base section of the large stem-loop structure. The stem-loop structures are predicted by the MFold program (Wisconsin GCG package) at 25°C and schematically drawn below the leader primary structures. The 5′- and 3′-sequences flanking the main structure are shown in open conformation. The stable structural element at the stem base (stem basal helix) and adjacent regions, are enlarged and their sequences shown. The nucleotide numbering is from the RNA 5′-end. The 5′-proximal short ORF (sORF1) is boxed. The sORF1 AUG and the non-AUG start codons in the shunt landing site are underlined. The identical nucleotide stretches/motifs in the shunt take-off and landing sites are highlighted in bold. Nucleotide substitutions that occur in five isolates of RTSV are indicated with arrows.

Translation of RTBV pgRNA is initiated by ribosome shunting that overcomes the obstacles of a 700-nt leader sequence with multiple sORFs and a stable stem-loop structure [8]. This mechanism operates efficiently in rice protoplasts and involves (i) 5′ end-dependent ribosome scanning until the first sORF is encountered, (ii) translation of this sORF and its termination just in front of the stem basal helix, the formation of which is crucial for efficient shunting, (iii) ribosome shunting over the structured region, and (iv) resumption of scanning at the shunt landing site, where a fraction of the shunting ribosomes (about 10%) also initiates translation at the AUU start codon of ORF I [8], [15] (Figure 1A). The RTBV shunt strikingly resembles the shunt mechanism evolved by *Cauliflower mosaic virus* (CaMV) from genus *Caulimovirus* of plant pararetroviruses [7], [15]. Notably, in both cases, initiation and proper termination of the first sORF translation (but not an encoded peptide) are essential for shunting. Furthermore, the RTBV shunt elements including the sORF, the stem base section and the shunt landing sequence could functionally replace the corresponding elements in the CaMV genome in driving efficient polycistronic translation of CaMV pgRNA and in supporting infection of the chimeric virus in CaMV-host plants [16].

The shunt configuration comprising an sORF terminating in front of the stable secondary structure has been identified in the pgRNA leader of most plant pararetroviruses [9], suggesting its evolutionary conservation within this family. Whether or not a shunt mechanism was also evolved in other families of plant viruses remained unknown so far. It is worth mentioning that an sORF-dependent shunt mechanism of the CaMV/RTBV-type has also evolved in a human spumavirus [17] and a human gene [18]. Here we provide evidence that sORF-dependent ribosome shunting operates in RTSV.

## Results/Discussion

### Identification of a conserved shunt configuration in the RTSV leader

Our computer-aided comparison of the 697-nt RTBV and the 514-nt RTSV leader sequences revealed remarkable similarities, suggesting that RTSV has co-evolved ribosome shunting (Figure 1):

1. Both leaders are unusually long, they contain several sORFs with a total number of 13 and 7 AUG codons, respectively, and can potentially fold into a stable stem-loop structure (deltaG = −217 and −194 kcal/mol, respectively). Although the RTSV structure is more branched, its bottom section is rich in GC base-pairs which would ensure the stability: indeed this section is present in all optimal and suboptimal structures predicted by MFOLD, whereas the upper part of the structure can potentially assume several different conformations (Figure S1, A and B). The primary sequences of the ascending and descending arms do not exhibit any apparent similarities between the two viruses. In CaMV, stability of the stem base but not primary sequences involved in its formation is an important parameter that determines shunt efficiency [15], [19]–[21]. Furthermore, in rice protoplasts the RTBV stem base section could be functionally replaced with the corresponding CaMV section composed of distinct primary sequences [15]. Moreover, a fully artificial stem structure placed downstream of a sORF could drive ribosome shunting *in vitro* [22]. According to our current shunt model, as compared to scanning ribosomes, the shunting ribosomes released after sORF translation have a reduced capacity to melt secondary structure and are therefore forced to resume scanning downstream of the structure [4], [10].
2. Both leaders have a very similar 5′-proximal sORF (sORF1). Firstly, sORF1 terminates at a short distance (7 and 8 nts, respectively) upstream of the stem base and a nucleotide context of the stop codon is identical (GAG UAG UCG). In CaMV and RTBV, the sORF1 stop codon is a take-off site for shunting ribosomes and proper termination of sORF1 translation in front of the stem base followed by peptide release is required for efficient shunting [4]. Secondly, the start codon of sORF1 is in a moderate initiation context in both RTBV and RTSV (UCA AUG GCU and CUU AUG GCC, respectively; the contexts deviate from a strong plant context because they lack A at position −3 relative to the first nucleotide of the start codon, but still have G at position +4) and it is positioned at a similar distance from the downstream secondary structure. Thirdly, the sORF1-encoded peptides are 6 and 5 amino acid long, respectively, and have identical termini: methionine and alanine at the N-terminus and glutamic acid at the C-terminus. Since the sORF1 amino acid composition generally does not affect shunt efficiency *in vitro* and *in planta* [15], [16], [20], [21], [23], the identity of terminal amino acids might reflect the importance of the nucleotide contexts surrounding the start and stop codon. The size of RTBV sORF1 may have become longer following the acquisition of ORF IV (a unique ORF, absent in closely-related badnaviruses) due to subsequent accommodation of an inefficient splice donor site within the sORF1 sequence in order to translate the sORF1-ORF IV fusion protein from spliced pgRNA [14].
3. In both leaders, the sequence downstream of the stem base (the landing site for shunting ribosomes in RTBV and CaMV) is UA-rich, which would ensure a low index of secondary structure. Unstructured nature of the shunt landing site is likely required for efficient resumption of scanning by shunting ribosomes. Furthermore, like in RTBV and CaMV, a presumptive shunt landing site in the RTSV leader contains a non-AUG start codon (AUA), which is located at a similar distance from the stem and followed by an additional in-frame non-AUG (AUU). Interestingly, both non-AUGs are in frame with the downstream AUG start codon of the polyprotein ORF, though unlike the RTBV AUU, the RTSV AUA and AUU codons are in suboptimal contexts. By analogy with RTBV, it is likely that one or both of these codons are inefficiently recognized by shunting ribosomes to initiate translation of N-terminally extended polyprotein. This hypothesis is further supported by an *in vitro* study of the CaMV shunt, in which two non-AUG codons located within the landing site were shown to initiate translation, albeit much less efficiently than the downstream AUG [23].
4. In both leaders, an identical stretch of pyrimidines (UUUCU) is located just downstream of the stem basal helix. By analogy with animal picornaviruses which contain a pyrimidine tract in their IRES elements just upstream of the initiation codon [5], it can be suggested that initiation at the non-AUG codon by shunting ribosomes might be facilitated by the UUUCU motif.

Thus, all the *cis*-acting elements known to drive ribosome shunting in RTBV are also present in RTSV, strongly supporting the idea that RTSV could have co-evolved an sORF-dependent shunt mechanism. Moreover, the identity of certain sequence motifs within these elements raises a possibility of their horizontal transfer from one virus to another during co-evolution. Alternatively, these motifs could have co-evolved independently through adaptation to the rice translational machinery.

### Translation downstream of the RTSV leader is initiated by ribosome shunting

To test the hypothesis that translation of RTSV genomic RNA is initiated by an sORF1-dependent ribosome shunting, we used well-established translation assays based on rice protoplasts and wheat germ extracts, in which translation of a reporter ORF encoding chloramphenicol acetyl transferase (CAT) placed downstream of the RTSV leader sequence or its mutant versions was monitored. We followed the same experimental settings and protocols as those used previously in a comparative study of molecular mechanisms of the RTBV and CaMV shunting [15].

**Figure 2:**
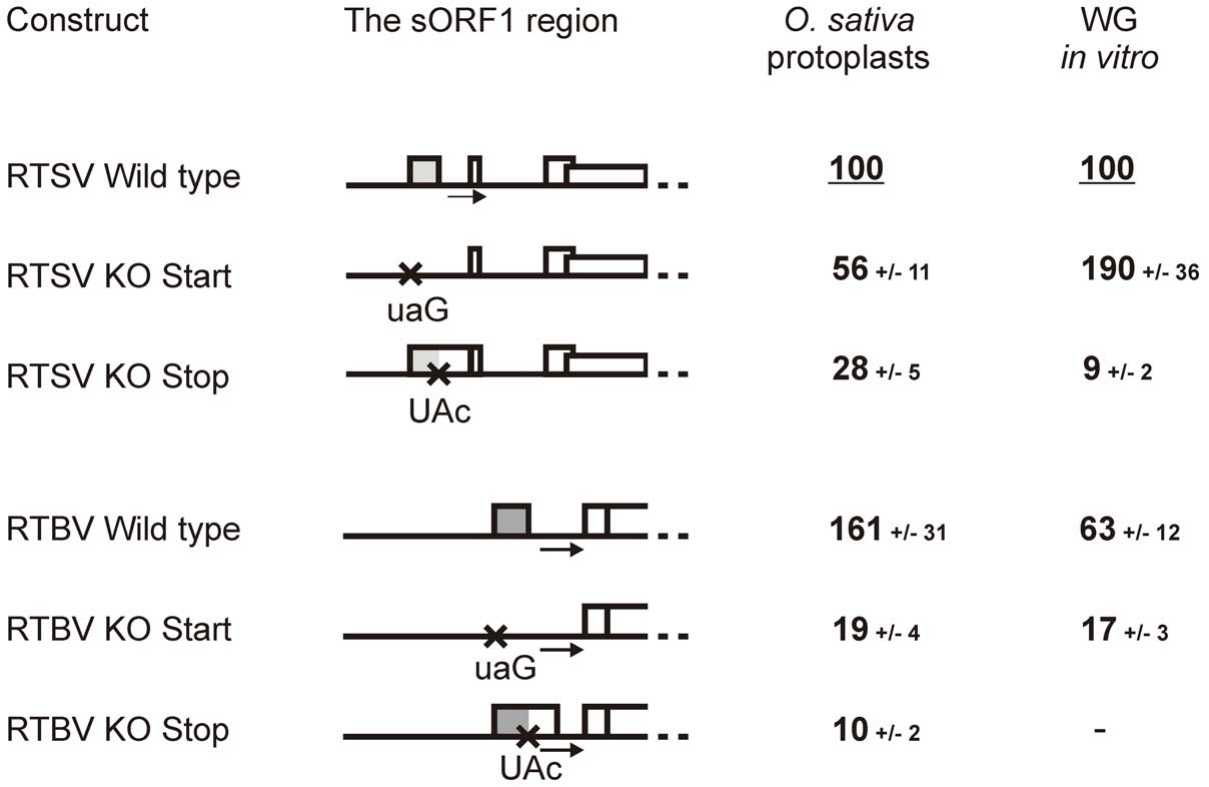
Translation downstream of the RTSV and RTBV leaders is regulated by the first sORF. Relative values of CAT expression downstream of the wild type and mutated versions (‘KO start’ and ‘KO stop”) of the RTSV (top panel) and RTBV (bottom panel) leaders in the two translation systems are given. Expression from the wild type RTSV construct in *O. sativa* (rice) protoplasts and in the wheat germ (WG) *in vitro* system is set to 100%. The sORF1 region of the leaders in each construct is shown schematically; point mutations are indicated with crosses and sORFs with boxes.

In rice protoplasts, both RTSV and RTBV leaders drove relatively efficient translation of the reporter ORF, although the RTBV leader allowed a 1.6-fold higher initiation rate. Confirming our previous results, knock out (KO) mutations of the start (AUG to UAG) or stop (UAG to UAC) codon of RTBV sORF1 drastically reduced translation (Figure 2). The same KO mutations of the RTSV sORF1 start or stop codons resulted in a significant decrease in downstream translation, albeit less dramatic than in the case of RTBV. This indicates that translation initiation downstream of the RTSV leader is sORF1-dependent, which is not consistent with internal ribosome entry at the 3′ end of the leader. Interestingly, the stop codon KO had a more pronounced effect by reducing the translation rate to 28%, whereas the start codon KO reduced translation only to 56% of the wild type level. This suggests that the RTSV leader lacking the first sORF AUG allows a relatively efficient linear ribosome migration towards the 3′ end, i.e. by leaky scanning through the remaining five AUGs and/or translation at some of the remaining five sORFs followed by reinitiation event(s). In CaMV, such a linear ribosome migration along the leader sequence has been investigated by mutating nine AUGs individually and in combinations and found to be 5 times less efficient than ribosome shunting in plant protoplasts [20] and wheat germ extracts [24]. In the case of RTBV, linear ribosome migration is even less efficient, likely because of a larger number of the intervening AUGs (twelve) and sORFs (eleven) (Figure 1). The KO of stop codon should not affect the initiation step of sORF1 translation but should result in termination of this translation event downstream of the shunt take-off site, which would diminish shunting but would not affect linear ribosome migration following sORF1 translation.

**Figure 3:**
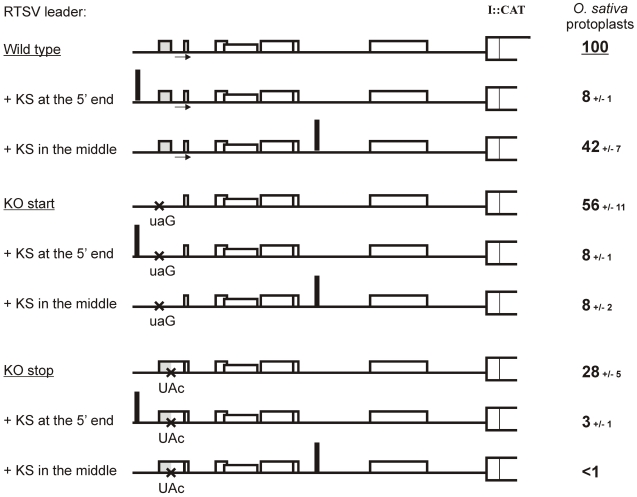
Translation downstream of the RTSV leader is initiated by shunting but not internal initiation. Relative values of CAT expression downstream of the wild type (“Wild type”) and sORF1-mutated versions (“KO start” and “KO stop”) of the RTSV leader carrying the KS at the 5′ end or in the middle region in *O. sativa* (rice) protoplasts are given. CAT expression from the wild-type leader construct in the absence of KS is set to 100%. For each construct, the RTSV leader preceding the polyprotein ORF (ORF I) fused to the CAT reporter ORF is depicted as thick line: the sORFs are indicated by boxes, point mutations shown with crosses, KS insertions indicated with thick vertical lines.

To further verify that sORF1-dependent translation downstream of the RTSV leader is initiated by ribosome shunting and evaluate a contribution of linear ribosome migration, we used a 40-nt Kozak-stem (KS) sequence which forms a perfect, compact stem-loop structure and blocks linear migration of scanning ribosomes [25]. In the case of RTBV and CaMV, insertion of KS in the leader region upstream of the first sORF abolished downstream translation, whereas its insertion within the leader region which is bypassed by shunting ribosomes had no dramatic effect on downstream translation [7], [8], [15], [20]. Likewise, insertion of KS at the 5′-end of the wild-type RTSV leader or its mutant versions with the sORF1 start or stop codon KO mutation nearly abolished downstream translation (Figure 3). This indicates that translation initiation in RTSV is 5′ end-dependent, thus ruling out internal initiation. Insertion of KS in the middle of the wild type RTSV leader did not abolish downstream translation, although the initiation rate was reduced to 42%. With KS inserted in the middle of the RTSV leader, KO mutation of either start or stop codon of sORF1 abolished downstream translation (Figure 3).

Taken together, we conclude that almost half of the ribosomes entering at the 5′ end of the RTSV leader and initiating translation of sORF1 are able to shunt over the structure and re-initiate translation at the 3′-end of the leader. Notably, like in CaMV and RTBV, this mechanism depends on proper termination of sORF1 translation in front of the structured region. Extension of RTSV sORF1 by the stop codon KO mutation should lead to termination at the in-frame stop codon located 10 triplets downstream, i. e. within the ascending arm of the structure. This would melt the stem basal helix and bring the terminating ribosome away from the take-off and landing sites.

**Figure 4:**
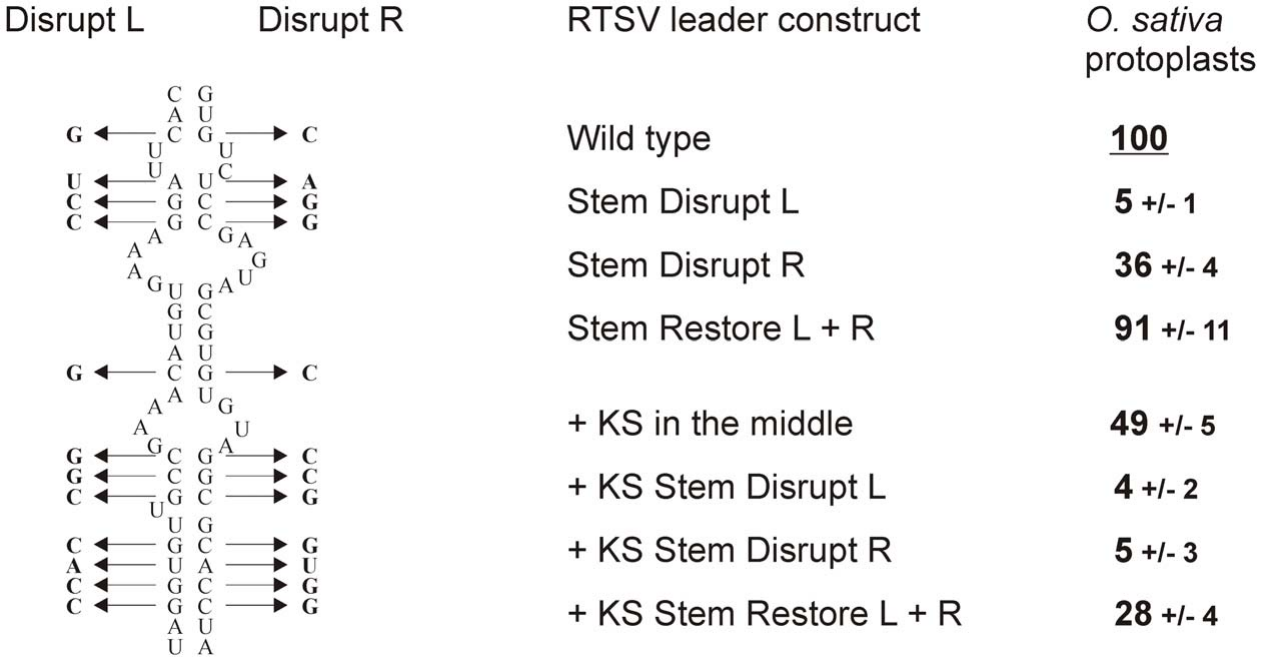
Integrity of the stem base secondary structure is essential for RTSV shunting. Twelve point mutations in either ascending (Disrupt L) or descending (Disrupt R) arm of the RTSV stem base secondary structure are shown on the left side. A combination of these mutations (Restore L+R) restores stable secondary structure. On the right side, relative values of CAT expression downstream of the wild type (“Wild type”) and the stem base-mutated versions (“Stem Disrupt L”, “Stem Disrupt R” and “Stem Restore L+R”) of the RTSV leader [or its variant with the Kozak stem (KS) sequence in the middle part] in *O. sativa* (rice) protoplasts are given. CAT expression from the wild-type leader construct is set to 100%.

To test if the stem basal helix structure is required for RTSV shunting, twelve point mutations were introduced either in its 5′-proximal or 5′-distal arms, which would disrupt secondary structure, and the compensatory mutations in both arms, which would restore stable secondary structure (Figure 4). The basal helix mutants with and without the KS sequence in the middle of the RTSV leader were constructed. Transient expression of the resulting constructs in rice protoplasts showed that disruption of the basal helix drastically reduced translation downstream of the RTSV leader, whereas restoration of the helix structure by the compensatory mutations almost fully restored downstream translation (Figure 4). We conclude that integrity of stable secondary structure but not primary sequences involved in formation of the stem basal helix is essential for ribosome shunting in RTSV.

Interestingly, in the absence of KS, the mutations in the 5′-proximal arm nearly abolished translation (5% of the wild type level), whereas the mutations in the 5′-distal arm reduced translation to 36% of the wild type level. The latter mutations in the presence of KS nearly abolished downstream translation (5% of the wild type level) (Figure 4). This suggests that, besides shunting, linear ribosome migration following translation of sORF1 is also abolished by the mutations in the primary sequence just downstream of sORF1. By contrast the mutations of the 5′-distal arm sequence located far away of sORF1 do not appear to affect linear ribosome migration, which would account for relatively high translation efficiency in this case, comparable to the translation efficiency of the RTSV constructs lacking sORF1. Notably, the negative effect of the 5′-proximal arm mutations is also evident when the RTSV basal helix is restored by compensatory mutations in the 5′-distal arm.

We have established previously that, in wheat germ extracts supporting efficient ribosome shunting driven by the CaMV shunt elements [23], [24], the RTBV shunting is about 7 times less efficient [15]. This is entirely due to incompatibility of the RTBV landing sequence, because the wheat germ translation machinery prefers A-rich rather than U-rich sequences and perhaps other unknown *cis*-elements present in the CaMV landing site but absent in the RTBV one [15]. Similar to RTBV, translation downstream of the RTSV leader was also relatively inefficient in wheat germ, although the RTSV leader allowed a 1.6-fold higher initiation rate (Figure 2). The KO mutation of sORF1 start codon increased downstream translation 1.9-fold. This is unlike RTBV, in which the sORF1 start codon KO reduced downstream translation about 4-fold (Figure 2). As discussed above, the RTSV leader allows much more efficient linear ribosome migration downstream of sORF1 than the RTBV leader, which explains a positive effect of the RTSV sORF1 start codon removal in the wheat system where shunt efficiency is diminished. KO mutation of the RTSV sORF1 stop codon abolished downstream translation in the wheat system (Figure 2). This shows that most of translation downstream of the RTSV leader depends on proper termination of sORF1 translation.

Taken together, we demonstrate here that translation initiation of RTSV genomic RNA is controlled by its long leader and mediated largely by sORF1- and stem basal helix-dependent ribosome shunting. Further research is needed to characterize this mechanism in more detail. But given the striking similarity of all the shunt elements in RTSV and RTBV and especially the identity of certain sequence motifs in the shunt take-off and landing sites, it is very likely that both RTSV and RTBV use a similar shunt mechanism.

### Conservation of the shunt *cis*-elements in RTSV isolates

Our comparison of five isolates of RTSV (NC_001632 and AM234048, AM234049, U71440, and AB064963) showed that the leader sequence is remarkably conserved with only 35 polymorphic positions including 33 single nucleotide substitutions and 1-nt and 2-nt insertions/deletions (not shown). Only four substitutions occur in the shunt elements – one in the shunt landing sequence between the pyrimidine stretch and the non-AUG codon and three in the stem basal helix primary sequence (but not in the secondary structure) (Figure 1B). Notably, in some regions downstream of the leader, RTSV sequences have a much higher polymorphism than the leader itself (not shown).

We conclude that the shunt elements are well preserved in all RTSV isolates, indicating their biological importance for the virus. When an infectious clone of RTSV becomes available it will be important to test the role of sORF1 and other *cis*-elements identified in this study for viral infectivity. Previously, it has been shown that sORF-dependent ribosome shunting is essential for infectivity of CaMV [21] and that the RTBV shunt elements can functionally substitute for the corresponding CaMV elements in systemic infection with a chimeric virus [16].

### RTSV may have acquired ribosome shunting after its encounter with RTBV

*Maize chlorotic dwarf virus* (MCDV), the second recognized member of genus *Waikavirus* [2], also possesses a long leader (434-nt in the type species NC_003626) with several sORFs (a total of 6 AUGs in NC_003626) and stable secondary structure (−154 kcal/mole in NC_003626; Figure S2). However, this leader sequence is highly polymorphic in three known isolates (less than 40% nucleotide identity) (data not shown). This suggests that a translation initiation mechanism may not be conserved. Interestingly, in all three isolates the first sORF in the MCDV leader is preserved in length (5 codons) but not nucleotide content. However, it terminates 145 nts upstream of the main structure in NC_003626 (Figure S2), which is not compatible with ribosome shunting. Furthermore, owing to high polymorphism, the shape, stability and position of the main structure are not preserved in MCDV isolates and the number and configuration of sORFs is also variable (data not shown). This again argues against shunting as the initiation mechanism. Nevertheless, the preservation of the first sORF suggests its importance in controlling translation initiation on MCDV genomic RNA which may occur via linear ribosome migration following translation of the first sORF. In support of this hypothesis, our above results for the RTSV leader indicate that in addition to ribosome shunting, sORF1-dependent linear ribosome migration also contributes to translation initiation downstream of the leader. It can therefore be proposed that in waikaviruses a linear ribosome migration-dependent mechanism has evolved earlier than shunting and that the ribosome shunt is a so-far unique acquisition by RTSV following its encounter with RTBV in a disease complex. However we cannot exclude an independent evolution of ribosome shunting in RTSV in the process of adaptation of the virus to the host plant translational machinery.

Among other viruses of the family *Secoviridae*, *Parsnip yellow fleck virus* (PYFV), the only recognized member of genus *Sequivirus*, is most closely related to RTSV and MCDV [2]. Unlike RTSV and MCDV, this virus has a shorter leader sequence (278 nts) that does not contain sORFs and cannot fold into stable secondary structure as predicted by MFOLD (data not shown). This suggests a linear scanning-dependent mechanism of translation initiation in PYFV. We cannot rule out, however, that PYFV (and MCDV) may use an internal initiation mechanism similar to that of potyviruses [5].

It is thought that the ribosome shunt mechanism in plant pararetroviruses has evolved in order to protect the viral coat protein-binding, secondary structure element located within the leader [26] – an RNA packaging signal – from being melted by linearly-migrating scanning ribosome [16]. A mechanism of packaging in RTSV is unknown: but conservation of the shunt mechanism between RTBV and RTBV raises a possibility that a packaging element may reside within the structured region of the RTSV leader.

## Materials and Methods

### Plasmid constructs

The RTBV leader constructs “Wild type” and “KO start” have been described earlier [15]. The RTSV leader construct “Wild type” (Figure 1) is a derivative of the corresponding RTBV construct, in which the RTSV genomic RNA sequence from position +1 till position +535 (i.e. the leader sequence followed with a 21-nt segment of the polyprotein ORF) was inserted between the CaMV 35S promoter and the CAT reporter ORF in place of the RTBV leader (as a PCR-amplified RTSV fragment flanked with Cla I and Xho I and cloned into the corresponding sites of the vector). Note that this construct contains the natural polyprotein ORF start codon in a strong initiation context followed by 6 codons of this ORF and the CAT ORF fused to these 7 codons lacks its own ATG. In the RTBV constructs the CAT ORF begins with its own ATG in a strong context, which is in frame with the upstream AUU initiation codon located in the RTBV shunt landing site [15]. Point mutations of the RTSV sORF 1 start (ATG to taG) or stop (TAG to TAc) codons were introduced by PCR-based mutagenesis, yielding constructs ‘KO start’ and ‘KO stop’, respectively. The Kozak-stem (KS) sequence was introduced at the 5′ end of RTSV leader by cloning of a pre-annealed, self-complementary oligonucleotide CGGGGGCGCGTGGTGGCGGCTGCAGCCGCCACCACGCGCCCC (the self-complementary KS sequence is underlined) into the Cla I site of the RTSV plasmids “Wild type”, “KO start” and “KO stop”. The KS sequence shown above was also introduced in the middle of the RTSV leader sequence (in place of a guanosine at position 255) by a PCR ligation method, similar to that which we described previously [15]. Note that this insertion does not disrupt the RTSV secondary structure except that one of its branches is extended by KS (Figure S1C).

The RTSV leader constructs “Stem Disrupt L”, “Stem Disrupt R” and “Stem Restore L+R” were obtained using 353 bp and 200 bp synthetic DNA fragments of the RTSV wild type construct that contain sequences from Cla I to EcoRV and from EcoRV to Xho I, respectively, each with 12 point mutations shown in Figure 4. These fragments were introduced into the RTSV wild type construct individually or in combination by a two fragment ligation method. Same mutations were also introduced in the above-described construct carrying the KS sequence in the middle part of the RTSV leader: in this case 262 bp and 330 bp synthetic DNA fragments of the RTSV+KS construct were used, which contain sequences from Cla I to Pst I (located in the KS sequence) and from Pst I to Xho I, respectively, each with the 12 point mutations.

For the *in vitro* translation experiments, the T7 promoter was introduced just upstream of the RTSV full-length leader and their variants with the sORF1 mutations by subclonning the Cla I-Sph I fragment from the RTSV plasmids “Wild type”, “KO start” and “KO stop” in place of the corresponding fragment of the T7 promoter-RTBV leader-CAT ORF plasmid described previously [15].

### Transient expression in rice protoplasts

Protoplasts from suspension culture of *O. sativa* were prepared and transfected with plasmid DNA by a polyethylene glycol method as described previously [8], [15]. Briefly, 0.6×10^6^ protoplasts were transfected with 10 µg CAT-expressing plasmid and 2 µg β-glucuronidase (GUS)-expressing plasmid or 5 µg green fluorescent protein (GFP)-expressing plasmid. The GUS or GFP plasmid served as an internal control of transfection efficiency. Following incubation for 19–24 hrs at 27°C in the dark, protoplasts were harvested, protein extracts prepared and assayed for CAT and GUS (or GFP) accumulation, as described previously [20]. Relative GUS activities were taken for normalization of the CAT expression levels given in Figure 2 and Figure 3, while relative GFP activities were taken for normalization of the CAT expression levels given in Figure 4. For each construct, the values given are the means of at least three experiments in independent batches of protoplasts. Deviations from the mean values generally did not exceed 20%. The levels of CAT mRNA accumulation were measured by quantitative RT-PCR with CAT ORF-specific primers using previously-described protocols for total RNA preparation, cDNA synthesis and real time PCR [27] and found to be comparable for all the RTSV constructs (data not shown).

### *In vitro* transcription and translation

The *in vitro* experiments were performed as described in detail earlier [15]. Briefly, the T7-promoter plasmids were linearized by Sph I and transcribed in the presence of the cap analog ^7^mGpppG (in 6-fold molar excess over GTP) by incubation with T7 RNA polymerase (Biofinex). The integrity of the synthesized transcripts was evaluated on a 6% denaturing polyacrylamide gel. Equimolar amounts of capped transcripts (0.5 pmol) were translated for 1 hour at 27°C in a wheat germ extract. Accumulation of CAT protein in translation mixture was measured in duplicate by CAT ELISA (Roche) as recommended by the manufacturer. For each construct, *in vitro* translation was performed at least three times with freshly prepared capped RNA, yielding similar results.

### Prediction of RNA secondary structure

Secondary structures at 25°C were predicted using the MFOLD program (Wisconsin Package, version 6.0; Genetics Computer Group, Madison, WI, USA). The most optimal and suboptimal secondary structures of the 515 nt RTBV leader sequence are shown in Figure S1. Folding of the RTSV leader sequence extended by either the natural RTSV coding sequence or the CAT reporter ORF sequence (present in the RTSV constructs tested here in the translational assays) did not affect the formation of the base section present in both optimal and suboptimal conformations (data not shown). Notably a free energy of the most optimal leader structure in RTSV (deltaG = −194.1 kcal/mol) is much more negative than that of fully randomized sequences of the same length (deltaG = ca. −100 kcal/mol; [28]).

MFOLD prediction of RNA secondary structure has proven to be reliable. For example, an MFOLD-predicted, large stem-loop structure of the 612-nt CaMV leader has been largely confirmed *in vitro* using chemical and enzymatic methods, though alternative conformations were also revealed in that study [29].

## Supporting Information

Figure S1 **The optimal and suboptimal structures of RTSV leader.** The optimal (**A**) and suboptimal (**B**) structures of the RTSV leader predicted by the Wisconsin GCG MFOLD are shown. Positions of the sORFs' start and stop codons are indicated in red and green, respectively. The stem basal helix is encircled. (**C**) The optimal structure of the RTSV leader with (on the left) and without (on the right) the Kozak-stem (KS) sequence insertion. The KS is encircled.
(PPTX)

Figure S2 **The optimal structure of MCDV leader.** The structure predicted by the GCG MFOLD is shown with positions of the sORF start and stop codons indicated in red and green, respectively.
(PPTX)
