# Supplementary material for: Short ORF-Dependent Ribosome Shunting Operates in an RNA Picorna-Like Virus and a DNA Pararetrovirus that Cause Rice Tungro Disease
Source: PLoS Pathog. 2012 Mar 1;8(3):e1002568. doi: 10.1371/journal.ppat.1002568 (PMC3291615; doi:10.1371/journal.ppat.1002568)
Supplement: Figure S1 — The optimal and suboptimal structures of RTSV leader. The optimal (A) and suboptimal (B) structures of the RTSV leader predicted by the Wisconsin GCG MFOLD are shown. Positions of the sORFs' start and stop codons are indicated in red and green, respectively. The stem basal helix is encircled. (C) The optimal structure of the RTSV leader with (on the left) and without (on the right) the Kozak-stem (KS) sequence insertion. The KS is encircled. (PPTX) [file ppat.1002568.s001.pptx]

## Slide 1
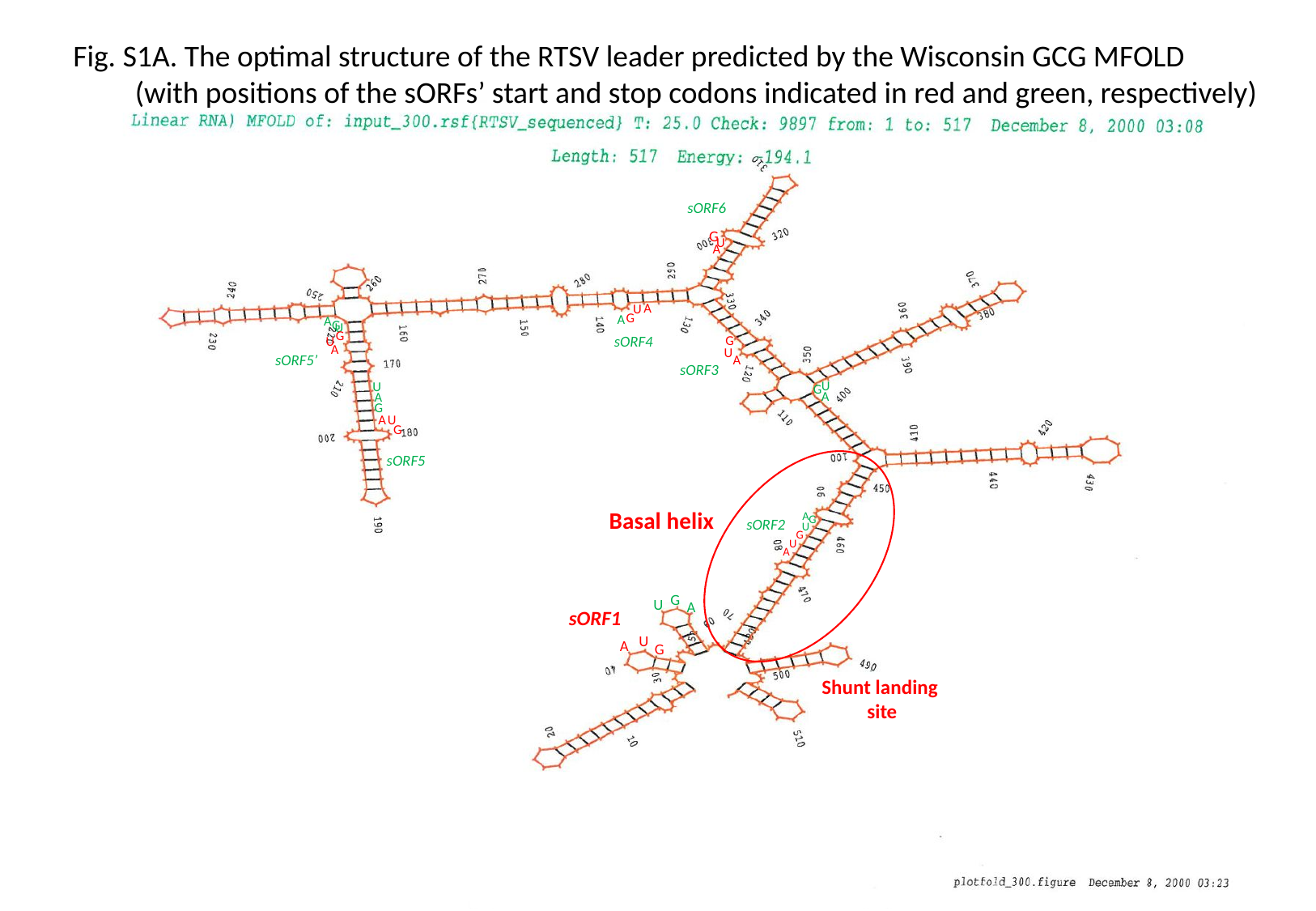

Fig. S1A. The optimal structure of the RTSV leader predicted by the Wisconsin GCG MFOLD
 (with positions of the sORFs’ start and stop codons indicated in red and green, respectively)
sORF6
G
U
A
A
U
G
A
A
G
U
G
sORF4
G
U
A
U
sORF5’
A
sORF3
U
U
G
A
A
G
U
A
G
sORF5
Basal helix
A
G
sORF2
U
G
U
A
G
U
A
sORF1
U
A
G
Shunt landing
site

## Slide 2
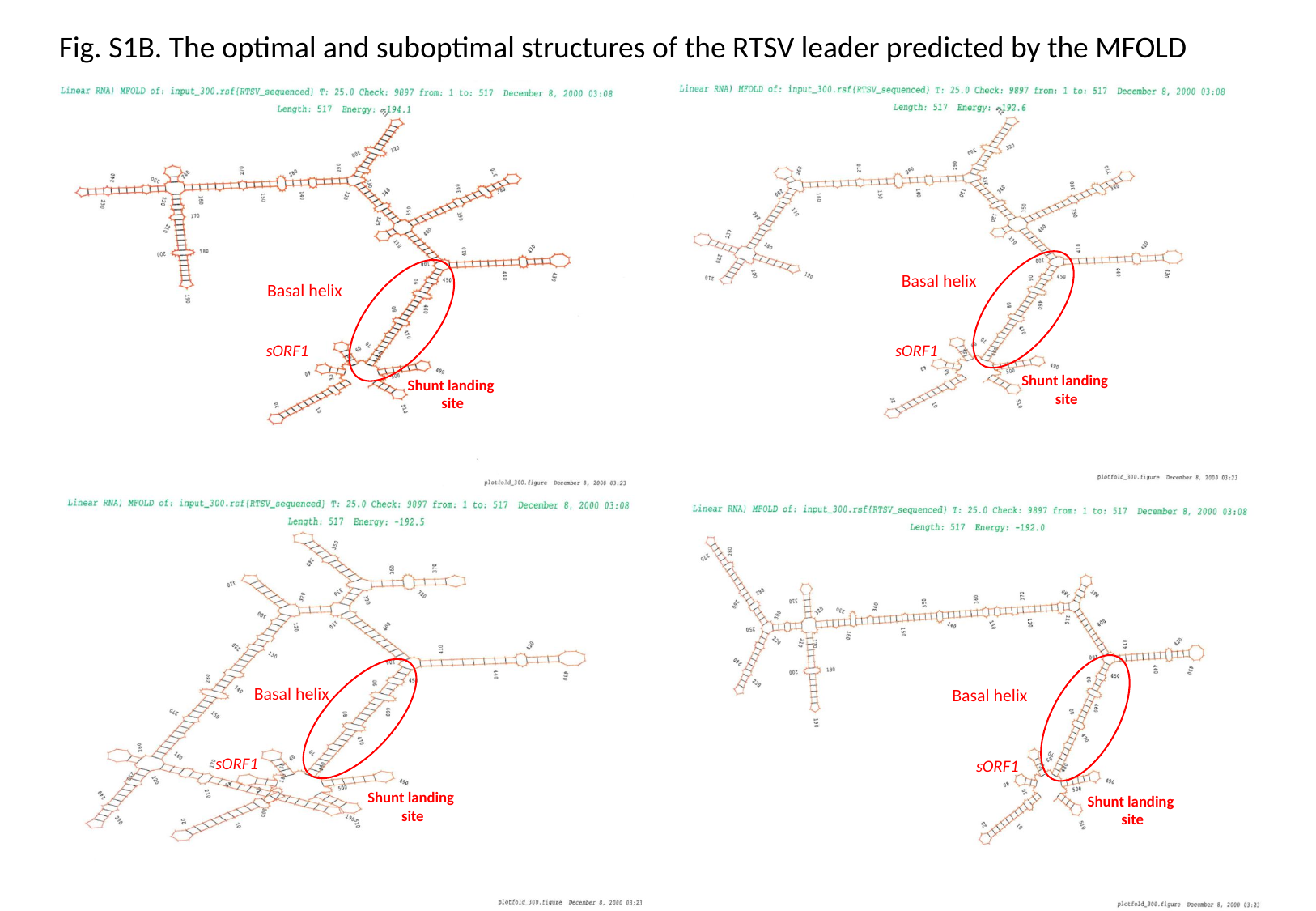

Fig. S1B. The optimal and suboptimal structures of the RTSV leader predicted by the MFOLD
Basal helix
Basal helix
sORF1
sORF1
Shunt landing
site
Shunt landing
site
Basal helix
Basal helix
sORF1
sORF1
Shunt landing
site
Shunt landing
site

## Slide 3
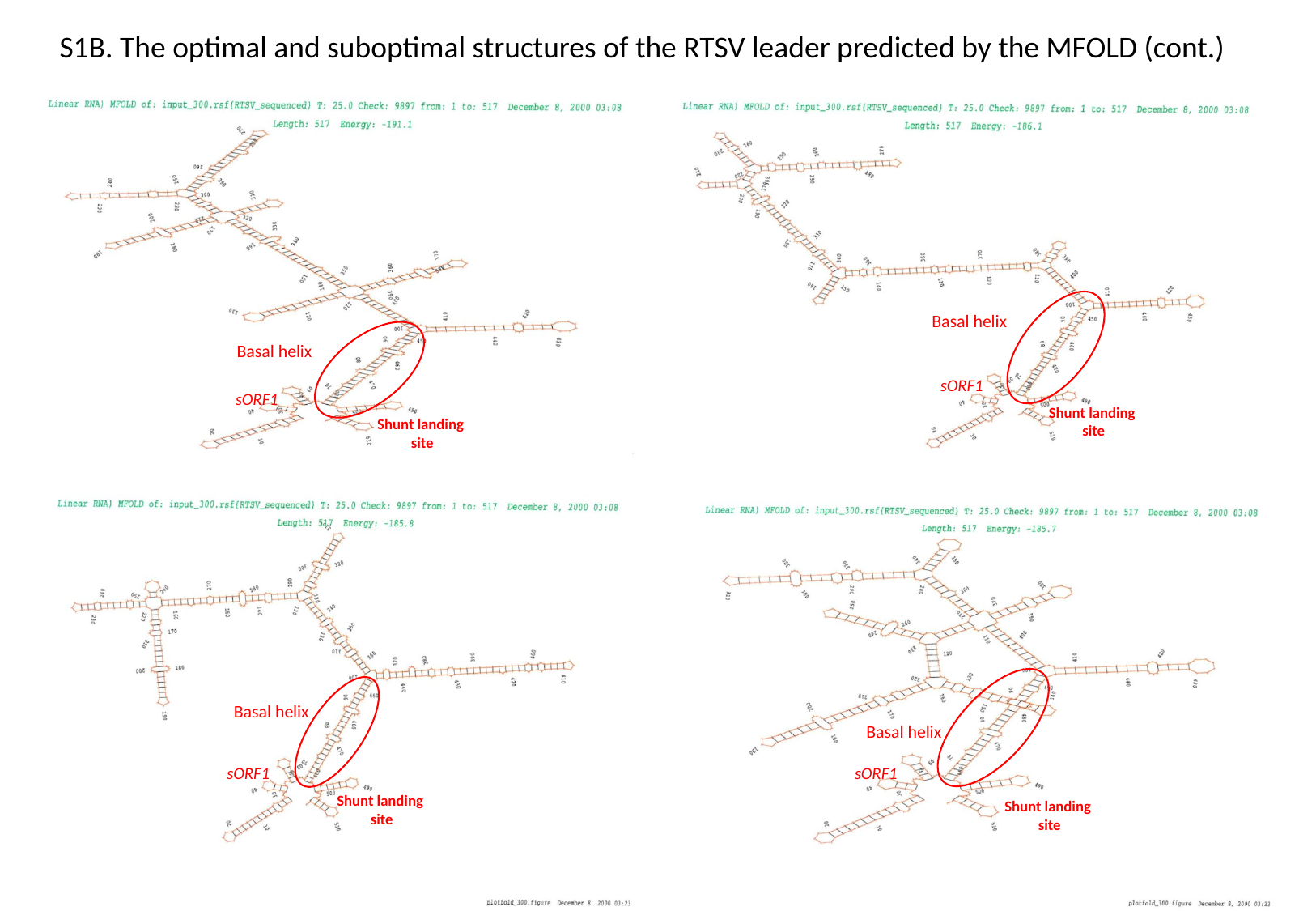

S1B. The optimal and suboptimal structures of the RTSV leader predicted by the MFOLD (cont.)
Basal helix
Basal helix
sORF1
sORF1
Shunt landing
site
Shunt landing
site
Basal helix
Basal helix
sORF1
sORF1
Shunt landing
site
Shunt landing
site

## Slide 4
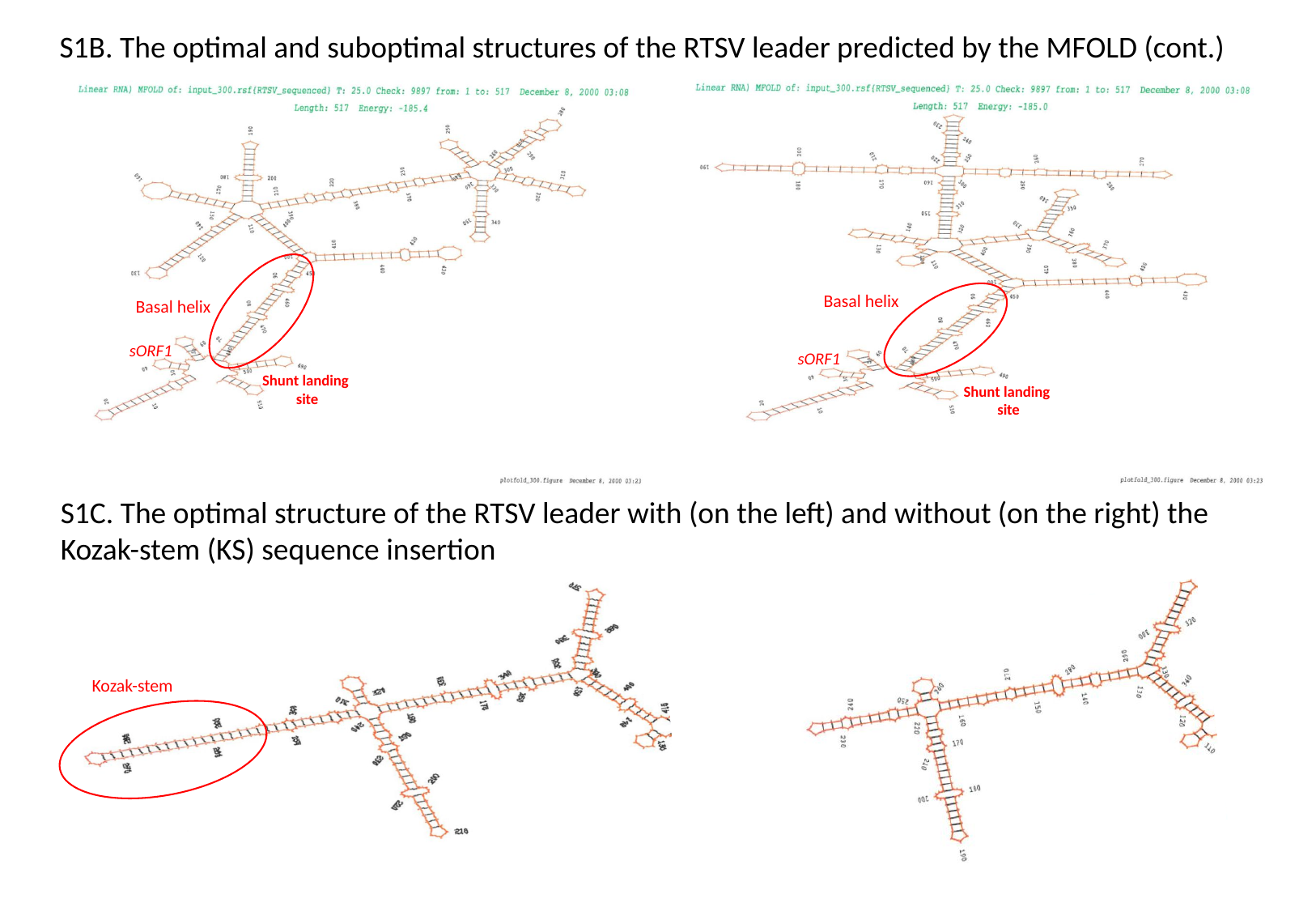

S1B. The optimal and suboptimal structures of the RTSV leader predicted by the MFOLD (cont.)
S1C. The optimal structure of the RTSV leader with (on the left) and without (on the right) the
Kozak-stem (KS) sequence insertion
Kozak-stem
Basal helix
Basal helix
sORF1
sORF1
Shunt landing
site
Shunt landing
site
