# Supplementary material for: Short ORF-Dependent Ribosome Shunting Operates in an RNA Picorna-Like Virus and a DNA Pararetrovirus that Cause Rice Tungro Disease
Source: PLoS Pathog. 2012 Mar 1;8(3):e1002568. doi: 10.1371/journal.ppat.1002568 (PMC3291615; doi:10.1371/journal.ppat.1002568)
Supplement: Figure S2 — The optimal structure of MCDV leader. The structure predicted by the GCG MFOLD is shown with positions of the sORF start and stop codons indicated in red and green, respectively. (PPTX) [file ppat.1002568.s002.pptx]

## Slide 1
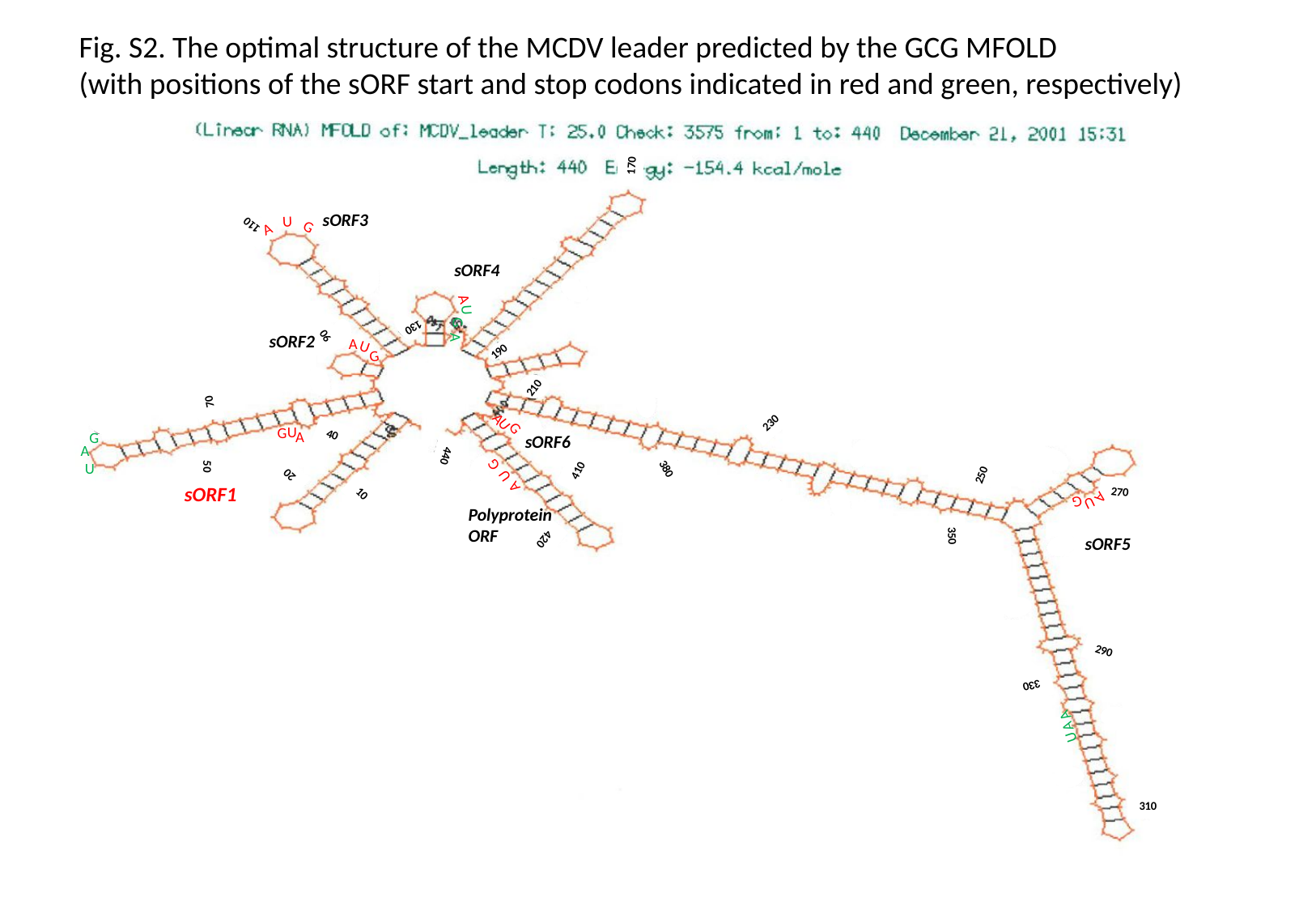

Fig. S2. The optimal structure of the MCDV leader predicted by the GCG MFOLD
(with positions of the sORF start and stop codons indicated in red and green, respectively)
U
G
A
G
A
U
sORF1
170
sORF3
U
G
110
A
sORF4
A
U
G
130
A
90
sORF2
A
U
190
G
210
70
A
U
230
G
40
sORF6
440
G
50
380
410
U
250
20
A
270
10
A
G
U
Polyprotein
ORF
350
sORF5
420
290
330
A
A
U
310
